# Anatomical insights into the vascular layout of the barley rachis: implications for transport and spikelet connection

**DOI:** 10.1093/aob/mcae025

**Published:** 2024-02-26

**Authors:** Twan Rutten, Venkatasubbu Thirulogachandar, Yongyu Huang, Nandhakumar Shanmugaraj, Ravi Koppolu, Stefan Ortleb, Götz Hensel, Jochen Kumlehn, Michael Melzer, Thorsten Schnurbusch

**Affiliations:** Leibniz Institute of Plant Genetics and Crop Plant Research, 06466 Gatersleben, Germany; Leibniz Institute of Plant Genetics and Crop Plant Research, 06466 Gatersleben, Germany; Leibniz Institute of Plant Genetics and Crop Plant Research, 06466 Gatersleben, Germany; Leibniz Institute of Plant Genetics and Crop Plant Research, 06466 Gatersleben, Germany; Leibniz Institute of Plant Genetics and Crop Plant Research, 06466 Gatersleben, Germany; Leibniz Institute of Plant Genetics and Crop Plant Research, 06466 Gatersleben, Germany; Leibniz Institute of Plant Genetics and Crop Plant Research, 06466 Gatersleben, Germany; Center for Plant Genome Engineering, Institute of Plant Biochemistry, Faculty of Mathematics and Natural Sciences, Heinrich Heine University Düsseldorf, 40225 Düsseldorf, Germany; Leibniz Institute of Plant Genetics and Crop Plant Research, 06466 Gatersleben, Germany; Leibniz Institute of Plant Genetics and Crop Plant Research, 06466 Gatersleben, Germany; Leibniz Institute of Plant Genetics and Crop Plant Research, 06466 Gatersleben, Germany; Institute of Agricultural and Nutritional Sciences, Halle-Wittenberg Faculty of Natural Sciences III, Martin Luther University, 06120 Halle, Germany

**Keywords:** Barley, *Hordeum vulgare*, rachis, spikelet, vasculature

## Abstract

**Background and Aims:**

Vascular patterning is intimately related to plant form and function. Here, using barley (*Hordeum vulgare*) as a model, we studied the vascular anatomy of the spike-type inflorescence. The main aim of the present work was to clarify the relationship between rachis (spike axis) vasculature and spike size, to define vascular dynamics and to discuss the implications for transport capacity and its interaction with the spikelets.

**Methods:**

We used serial transverse internode sections to determine the internode area, vascular area and number of veins along the rachis of several barley lines.

**Key Results:**

Internode area and total vascular area show a clear positive correlation with spike size, whereas the number of veins is only weakly correlated. The lateral periphery of the rachis contains large mature veins of constant size, whereas the central part is occupied by small immature veins. Spikelet-derived veins entering the rachis often merge with the immature rachis veins but never merge with the mature veins. An increase in floret fertility through the conversion of a two-rowed barley into an isogenic six-rowed line, in addition to a decrease in floret fertility owing to enhanced pre-anthesis tip degeneration caused by the mutation *tip sterile 2.b* (*tst2.b*), significantly affected vein size but had limited to no effects on the number of veins or internode area.

**Conclusions:**

The rachis vasculature is the result of a two-step process involving an initial layout followed by size adjustment according to floret fertility/spike size. The restriction of large mature vessels to the periphery and that of small immature vessels to the centre of the rachis suggests that long-distance transport and local supply to spikelets are spatially separated processes. The identification of spikelet-derived veins entering the rachis without fusing with its vasculature indicates that a vascular continuity between rachis and spikelets might be non-essential.

## INTRODUCTION

In cereals, the vasculature of the rachis varies considerably from that of the shoot. Percival (1921) stressed that in wheat (*Triticum aestivum*), the shoot displays a consistent shape and vascular organization throughout, whereas the corresponding features of the rachis change from semicircular to spindle-shaped within several internodes. The same author also suggested an acropetal decline in vascular number within the rachis. In barley (*Hordeum vulgare*), Kirby and Rymer (1975) discerned three types of vascular bundles comprising two opposite crescent rows of median veins demarcated by two large lateral veins, the whole of it flanked by a variable number of small peripheral veins. They reported further that at each rachis node, the two lateral veins and the whole arch of median veins adjacent to the spikelet attachment site divide, with one branch entering the spikelets and the other proceeding along the rachis. The median veins opposite the spikelets, however, do not divide but continue to the next rachis node. Studying only spike fragments, Kirby and Rymer (1975) refrained from commenting explicitly on an acropetal decline in vascular bundles. Whingwiri *et al.* (1981) confirmed the existence of a gradual acropetal decline in both the number of veins and their size in wheat spikes. Similar to Evans *et al.* (1970), they noted a striking near 1:1 relationship between the number of spikelets and the number of main vascular bundles at the base of the rachis. The idea of each spikelet being assigned to a singular vascular bundle, first expressed by Evans *et al.* (1970), was slightly revised into a model in which each spikelet is supplied by a constant number of one or two bundles (Whingwiri *et al.*, 1981). In line with this, Bremner (1972) concluded that wheat spikelets probably have their supporting vascular bundles linked in parallel, whereas the kernels within the spikelet are connected in series.

The simplicity of the ‘one vein for one spikelet’ model is appealing but hardly applicable to barley, in which the number of spikelets can easily exceed the number of rachis veins by a factor of three or more. A gradual acropetal decline in vein size, as reported for wheat, also has profound physical implications, because it will greatly increase the resistance to assimilate transport towards the apex (Chavarria and Pessoa dos Santos, 2012). The phenomenon of pre-anthesis tip degeneration in barley spikes and wheat spikelets, which is the failure of the most apical spikelets/florets to develop into grains (Appleyard *et al.*, 1982; Alqudah and Schnurbusch, 2014; Boussora *et al.*, 2019; Thirulogachandar and Schnurbusch, 2021), has sometimes been suggested to be a result of transport limitations caused by increased resistance (Kirby and Faris, 1970; Hanif and Langer, 1972; Whingwiri and Kemp, 1980). Nevertheless, when Bremner and Rawson (1978) removed the basal grains of wheat spikelets, they observed an improved growth rate of the more distal grains, suggesting that the limiting factor is not transport capacity but the availability of resources. Subsequent spikelet and grain removal experiments in rice (Kato, 2004; You *et al.*, 2016) and wheat (Ma *et al.*, 1996) confirmed these observations. Whingwiri *et al.* (1981) considered it premature to judge the vasculature of the spike to be a static entity. In wheat, Evans *et al.* (1970) noticed a clear correlation between the number of spikelets and both the size and number of vascular strands within the peduncle. This indicates the presence of a dynamic component in vascular development, which, according to Whingwiri *et al.* (1981), probably exerts itself in the early stages of spike development. Waddington *et al.* (1983) suggested the existence of two distinct dynamic components. The first is the developmental progress that ends when maximum yield potential is reached, here defined as the maximum number of spikelet primordia on a spike (Thirulogachandar and Schnurbusch, 2021). After this there is a phase of exponential growth of the initiated organs whose fates are determined before anthesis. Accordingly, the layout of the main rachis vasculature should be completed by the time of maximum yield potential and its final dimensions fixed before the start of anthesis.

Trying to shed more light on the vascular organization of the barley rachis, we investigated parameters including internode area, number of internodes and size of the main vascular bundles, in addition to their distribution within serial transverse internode sections obtained from mature spikes of the two-rowed cv. Golden Promise (two-rowed GP). The influence of spikelet fertility on rachis vasculature was studied in an isogenic six-rowed GP*-vrs1* line generated by targeted mutagenesis (Thirulogachandar *et al.*, 2024), and for the effect of a reduction in fertile spikelets, we compared the cv. Bowman with its near-isogenic mutant BW*-tst2.b* (Huang *et al.*, 2023) exhibiting extended pre-anthesis tip degeneration. Our results confirm the assumption of Waddington *et al.* (1983) that the morphogenesis of the barley rachis vasculature is the result of two distinct processes: initiation and processing, with ultimate vein size being strongly correlated with the number of fertile spikelets. Mature veins of constant size are located in the rachis periphery, whereas immature veins of declining size occupy the middle part, suggesting a spatial separation between long-distance transport and local delivery to the spikelets. Our data also indicate that, although spikelet-derived veins can enter the rachis both in the outer periphery and in the central part, they are only capable of fusing with the immature rachis veins residing in the centre.

A comparative analysis of the wild barley accession HID003 revealed a similar vascular layout with slight differences in the vascular dynamics.

## MATERIALS AND METHODS

### Plant material

The two-rowed barley (*Hordeum vulgare* L.) cv. Bowman and cv. Golden Promise (two-rowed GP) and wild barley [*H. vulgare* L., subsp. *spontaneum* (K. Koch)] accession HID003 were obtained from the IPK gene bank. The barley mutant, *tst2.b* (BW883), was ordered from the NordGen seed bank. To reduce background introgressions from the original mutagenesis recipient (cv. Donaria), we further backcrossed BW883 to wild-type Bowman plants (Huang *et al.*, 2023). The isogenic six-rowed GP*-vrs1* line was obtained through targeted mutagenesis conducted by CRISPR-associated (Cas) endonuclease technology (Thirulogachandar *et al.*, 2024). A guide RNA (TCTGGAGCTGAGCTTCCGGGAGG) was designed for the homeodomain region of the *Vrs1* gene and inserted between the OsU3 promoter and the downstream gRNA scaffold present in a generic, monocot-compatible intermediate vector, pSH91 (Budhagatapalli *et al.*, 2016). Next, the whole expression cassette of the gRNA-Cas9 was introduced into the SfiI cloning site of the binary vector p6i-d35S-TE9 (DNA-Cloning-Service, Hamburg, Germany). Transgenic plants were created in the two-rowed cv. Golden Promise, via *Agrobacterium*-mediated stable transformation (Hensel *et al.*, 2009). The two-rowed GP and its gene-edited six-rowed GP*-vrs1* were grown in 14 cm pots; cv. Bowman, line BW*-tst2.b* and wild barley accession HID003 were grown in 9 cm pots. Plants were cultivated in greenhouse conditions at 22/18 °C in 16 h/8 h day/night.

### Rachis vasculature measurements

Spikes were collected after anthesis, by which time the development and growth of the barley spike, including its vasculature, is fully completed (Waddington *et al.*, 1983; Yang and Zhang, 2006). Spikes were usually collected from prime tillers (for an overview of plant material, see Table 1). In case of two-rowed GP, mature spikes were also collected from secondary tillers to obtain a larger variation in spike size. Starting with the peduncle, which was sampled 2 cm underneath the base of the rachis, and ending with the first internode of the zone of pre-anthesis tip degeneration, free-hand transverse sections were made at about one-third of the height of an internode measured from the base. Sections were examined in a Zeiss LSM780 laser scanning microscope (Carl Zeiss, Jena, Germany) using a ×10 NA 0.45 objective (zoom 1, image size 1024 × 1024 pixels combined with tiling and *z*-stacks with ensuing maximum intensity projection). Sectioned internodes were scanned with a 405 nm laser line, and cell wall autofluorescence was recorded with a 406–500 nm bandpass. For area measurements, the open-source Fiji software (Schindelin *et al.*, 2012) was used. The size of individual veins was determined as the area surrounded by a bundle sheath. Classical Student’s *t*-tests were performed to reveal significant differences between data sets.

**Table 1.** Overview of plant material used.

Plant material				
Plant	Sample size	Spike size (in nodes)	Average size (in nodes)	Internodes sectioned
Cultivar Golden Promise	14	30–45	36.5 ± 2.9	All
	41	25–45	347 ± 4.3	Internode 8
	Total: 55	25–45	35.2 ± 4.1	–
GP-*vrs1*	5	36–38	37.2 ± 0.7	All
Cultivar Bowman	11	23–27	25.3 ± 1.8	All
Bw-*tst2.b*	13	13–18	14.2 ± 1.8	All
Accession HID003	8	20–24	22.0 ± 1.4	All

## RESULTS

### Basic morphology of the barley rachis

**Fig. 1.**
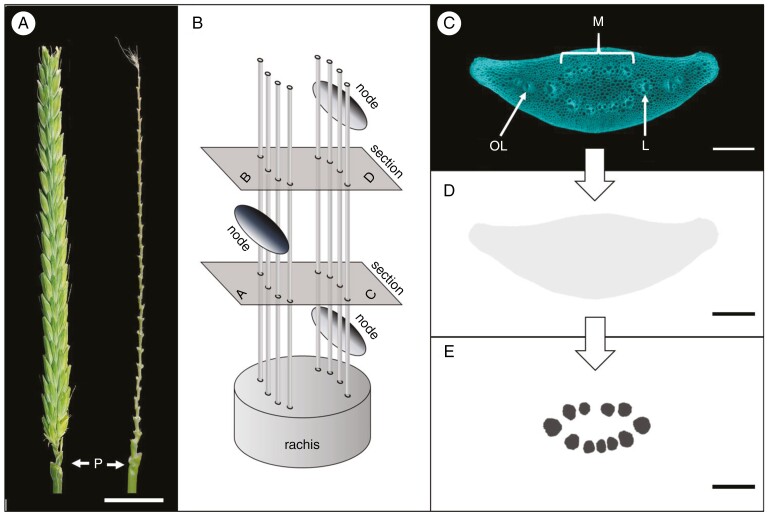
Barley cv. Golden Promise. (A) Mature spike and central view of rachis after removal of spikelets. Abbreviation: P, peduncle. (B) Schematic view of rachis showing the two opposite rows of median veins and the alternating arrangement of nodes on either side of the rachis. Serial internode sectioning enables the analysis of vascular changes that happen after passing a node (from A to B = internodal) and between two nodes (from C to D = intranodal). (C) Transverse internode section reveals three types of vascular bundles: median (M), lateral (L) and outer lateral (OL). (D) Schematic view of internode area in grey. (E) Schematic view of the size and distribution of main vascular bundles. Scale bar: 1000 µm (A), 200 µm (C–E).

To create a reference model for the shape and vascular organization of the barley rachis, we used fully differentiated spikes of two-rowed GP (Fig. 1A), with the number of nodes varying between 30 and 43 (average 36.5 ± 2.9 nodes, *n* = 14). By using serial transverse internode sectioning, each vein on its course between two nodes is cut both shortly after and shortly before accessing a node (Fig. 1B). The persistent morphological features of individual veins allow them to be followed throughout the rachis. The rachis starts as a semicircular cylindrical structure that rapidly attains an ellipsoid shape (Supplementary Data Video). The peduncle at the base of the rachis displays two rings of vascular bundles, of which the outer ring of smaller bundles gives rise to the outer lateral veins mentioned by Kirby and Rymer (1974) (Fig. 1C). The inner ring of larger bundles develops into the main rachis vasculature consisting of two opposing crescent rows of median veins with two large lateral veins at the vertices (Fig. 1C; Supplementary Data Video). Owing to their different origin and different morphological features, including much smaller metaxylem vessels, the outer lateral bundles were excluded from the present study, which thus deals exclusively with the median and lateral veins (Fig. 1E). Unless stated otherwise, the data presented here were collected from 14 mature spikes of two-rowed GP in the late stages of grain filling (average size 36.5 ± 2.9 nodes, *n* = 14).

### Rachis parameters

**Fig. 2.**
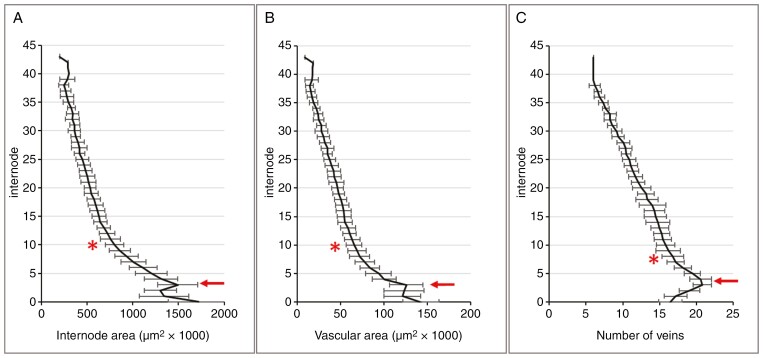
Dynamics of internode parameters along the rachis. (A, B) Internode area (A) and total vascular area (B) reach a local maximum at internode 3 (red arrow), followed by a steep decline until around internode 10 (*) and a much more gradual decline thereafter. (C) The number of veins peaks near internode 4 (arrow) before gradually declining towards the apex.

For a reconstruction of the vascular organization within the rachis, we first focused on the following parameters: internode area, total vascular area and number of vascular bundles. The internode area reaches a weak local maximum at internode 3 before decreasing acropetally (Fig. 2A). The fall-off is sharp before internode 10 but becomes gentler afterwards. The dynamic of the parameter total vascular area is remarkably similar to that of the internode area (compare Fig. 2A, B), including a small local maximum between internodes 2 and 3, followed by a decline, which is steep until about internode 10 and much more gradual thereafter. The third parameter, the number of veins, reaches an absolute peak at internode 3 or 4 before gradually declining towards the apex (Fig. 2C).

### Rachis parameters and spike size

**Fig. 3.**
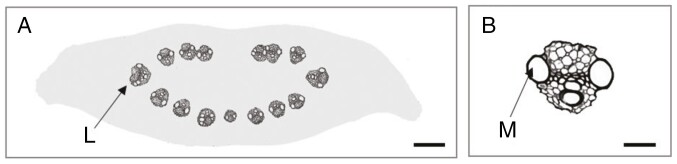
Schematic illustration of shape and vascular composition of internode no. 8 from a 36-node spike (A), with a lateral vein in detail (B). Abbreviations: L, lateral vein; M, metaxylem vessel. Scale bar: 100 µm (A) and 20 µm (B).

To determine the relationship between rachis parameters and spike size, a total of 55 spikes of two-rowed GP, with the number of nodes varying between 25 and 45, were used (average size 35.2 ± 4.1 nodes). From each of these spikes, internode 8 was collected, and the internode area, total vascular area, total lateral vein area and total area of the metaxylem vessels of the lateral veins (Fig. 3A, B) were measured.

**Fig. 4.**
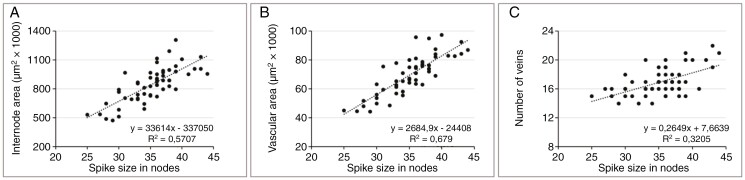
Rachis parameters in relationship to spike size. Data were collected for internode no. 8 isolated from a total of 55 spikes (average size 35.2 ± 4.1 nodes). Internode area (A) and total vascular area (B) show a clear positive correlation with spike size, whereas the number of veins (C) shows only a weak positive correlation.

**Fig. 5.**
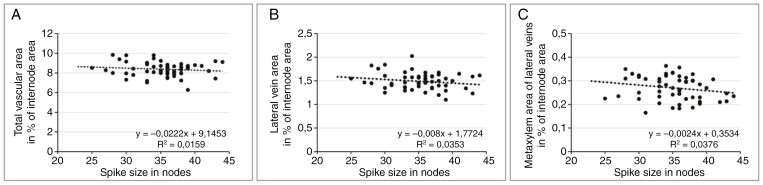
Vascular parameters in relationship to internode area. Data were collected for internode no. 8 isolated from a total of 55 spikes (average size 35.2 ± 4.1 nodes). Total vascular area (A), total lateral vein area (B) and total area of metaxylem vessels from the lateral veins (C) are given as a percentage of internode area in relationship to spike size. The results indicate that the ratios between internode area and vascular parameters down to metaxylem vessels are independent of spike size.

The results, presented as dot plots, demonstrate that the internode area (Fig. 4A) and total vascular area (Fig. 4B) show a clear positive correlation with spike size. In contrast to this, the number of veins displays only a weak positive correlation (Fig. 4C). The same data were then used to investigate the relationships among the vascular parameters themselves. The outcome indicates that within a given internode, there are fixed ratios between internode area and the vascular parameters total vascular area (Fig. 5A) and individual vein area (Fig. 5B) down to metaxylem area (Fig. 5C). Given that these ratios are independent of spike size, internode area can be used to compare relative differences in transport capacities between spikes of different sizes.

### Dynamics of individual vein orders

**Fig. 6.**
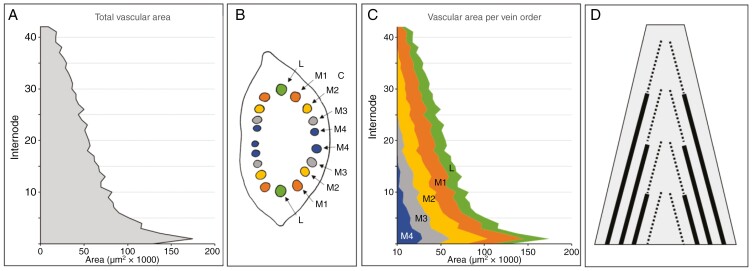
Contribution of individual vein orders to the overall vasculature along the rachis of a 43-node barley spike. (A) Total vascular area along the rachis. (B) Schematic overview of vascular organization in the second internode. Inwards of the lateral veins (L), the median veins are separated into first (M1), second (M2), third (M3) and fourth (M4)-order median veins. (C) Differentiating the vascular area per vein order reveals the sequential diminishing and ultimate disappearing of fourth (M4), third (M3) and second (M2)-order median veins towards the apex. (D) Schematic presentation of vascular layout in the barley rachis, with stably sized mature veins (solid lines) in the lateral periphery and a staggered array of diminishing immature veins (dotted lines) in the central area.

Treating the rachis vasculature as a whole masks the behaviour of its individual components. This is exemplified by a differentiated representation of the vascular bundles in a single 43-node rachis (Fig. 6). The graph for the total vascular area shows the characteristic peak at internode 3, a sharp decline until about internode 10, and a subsequent smooth decline (Fig. 6A). To distinguish the contributions of individual veins, the median vasculature was divided into veins of ascending order (Fig. 6B). Here, we followed the proposition of Kirby and Rymer (1975) that consecutive new classes of median veins develop inwards of pre-existing ones. Inwards of the lateral veins thus follow several orders of median veins, each comprising four vascular bundles (Fig. 6B). For clarity, all veins beyond third order were grouped as fourth-order veins. The results show that all vein orders initially mimic the dynamics of the total vascular area in showing a peak value around internode 2 followed by a brief steep decline (Figs 2 and 6C). The vascular area of fourth-order veins displays a continuous decline and vanishes around internode 15, whereas the other vein orders show increasingly long zones of size stability and do not start their final decline before the preceding vein order has disappeared (Fig. 6C, D). Accordingly, the middle part of the rachis is thus occupied by a staggered array of declining, probably immature veins, while stable-sized, probably mature, veins occupy the lateral regions (Fig. 6D).

### Decline in number of veins along the rachis

**Fig. 7.**
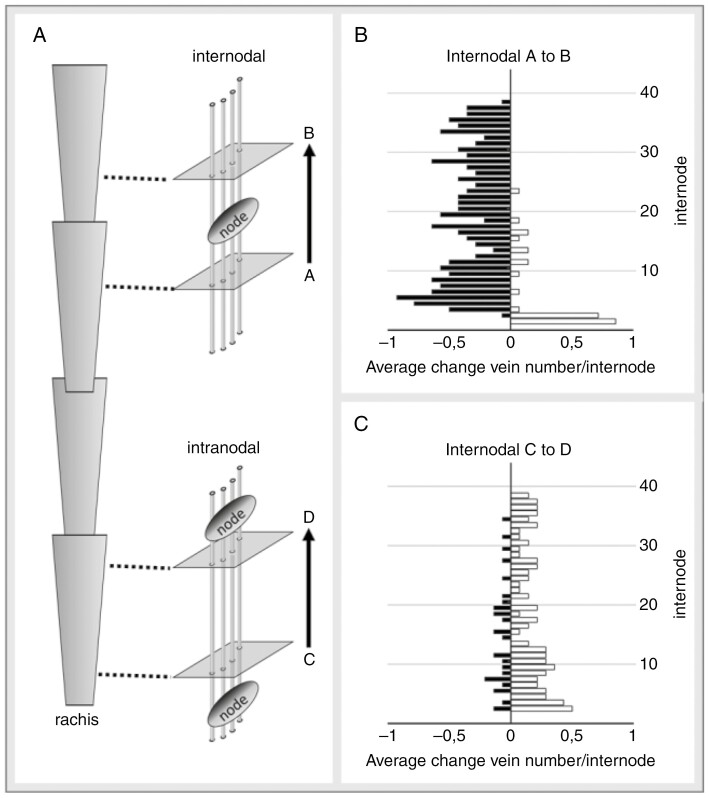
Internodal and intranodal changes in the number of veins along the rachis of mature spikes (average size 36.5 ± 2.9 nodes, *n* = 14). (A) Schematic view of measuring internodal and intranodal changes by serial internode sectioning. (B) Internodal changes start with a small zone of strong increases in the number of veins (open bars) at the extreme base of the rachis. After a tipping point at internode 3, the number of veins decreases (solid black bars) until the very tip of the spike. (C) Changes in the intranodal number of veins are less frequent than intranodal changes, lack an anomaly at the extreme base and are dominated by small but consistent increases in the number of veins (open bars).

The model for rachis vascular organization proposed in Fig. 6D predicts that rachis veins will ultimately fade into oblivion. If vein terminations are random, they should occur with similar regularity both internodal (i.e. after passing a node; Fig. 7A) and intranodal (i.e. between two nodes; Fig. 7A). However, the summation of the positive and negative changes in the number of veins along the rachis reveals a strong disparity between internodal (Fig. 7B) and intranodal changes in the number of veins (Fig. 7C). Internodal changes start with a strong increase in the number of veins at the extreme base until internode 3, then transform into a consistent decline running at an average of close to 0.4 veins per internode (Fig. 7B). In contrast to this, intranodal changes are far less common, do not show an anomaly at the extreme base and, most significantly, are dominated by small increases in the number of veins (Fig. 7C). The results thus suggest that most rachis veins ultimately end near or at a node. Furthermore, following the assumption that rachis veins do not merge or split within an internode (Kilby and Rymer, 1974), the small but consistent intranodal increase in the number of veins indicates that elements of the spikelet vasculature are entering and growing downwards into the rachis.

### Extra-numeral veins and rachis vasculature

**Fig. 8.**
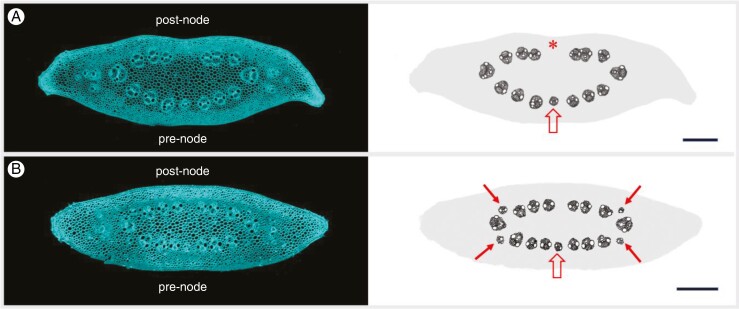
Presence of extra-numeral veins. (A) Internode no. 10 of a 36-node spike and schematic illustration of vascular distribution. The crescent row of median veins immediately apical to a node (post-node) reveals a conspicuous large empty area in the centre (*). In the opposite row of veins, which are about to reach a node (pre-node), the centre is occupied by a notable small vein (open arrow). (B) Internode no. 14 of a 35-node spike and schematic illustration of vascular distribution. Veins that are distinctly smaller than their neighbours are found in the pre-node centre (open arrow) and at all positions in between and slightly outside of the lateral and first-order median veins (arrows). Scale bars: 200 µm.

Putative spikelet-derived veins within the rachis expose themselves by what we refer to here as extra-numeral veins. This type of vein is usually distinctly smaller than its neighbouring counterparts. Extra-numeral veins can occasionally be identified in the centre of the rachis, occupying an otherwise void space within the crescent row of median veins (Fig. 8A). The most exposed group of extra-numeral veins, however, is found in the periphery of the rachis, where they are located in between but slightly outside of the much larger lateral and first-order median veins (Fig. 8B). The peripheral setting indicates that extra-numeral veins probably descend from the lateral spikelets.

**Fig. 9.**
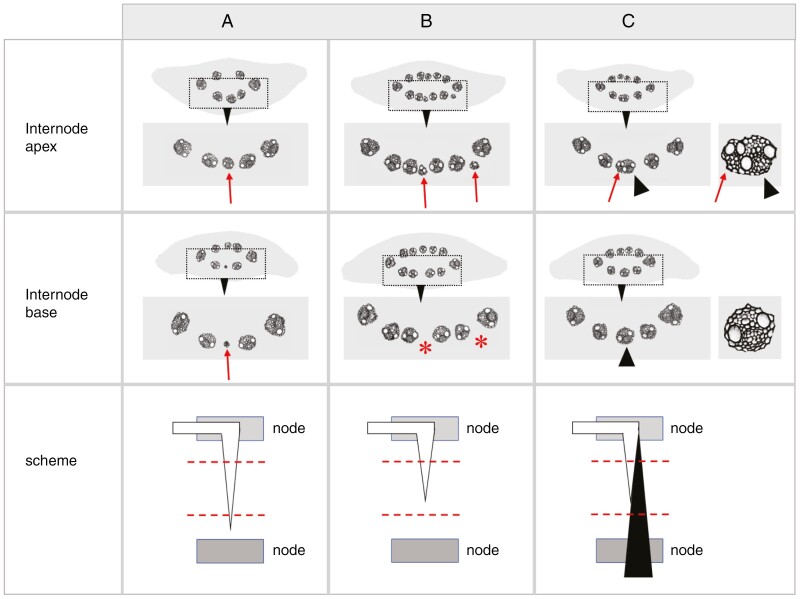
Identification of spikelet-derived extra-numeral veins in paired internode sections. Red arrows indicate extra-numeral veins basipetally decreasing in size (A), disappearing (*) before reaching the next node (B) or fusing with a rachis vein (black arrowhead) (C). Schematic views show extra-numeral veins in open black and rachis veins in solid black, with dashed red lines indicating the planes of sectioning.

Analysis of dual consecutive internode sections suggests that upon entering the rachis, extra-numeral veins continue in a basipetal direction. They rapidly decrease in size (Fig. 9A) and often disappear before reaching the next node (Fig. 9B). Extra-numeral veins that enter the central rachis can fuse with the local rachis vasculature (Fig. 9C).

### Floret fertility and rachis vasculature

**Fig. 10.**
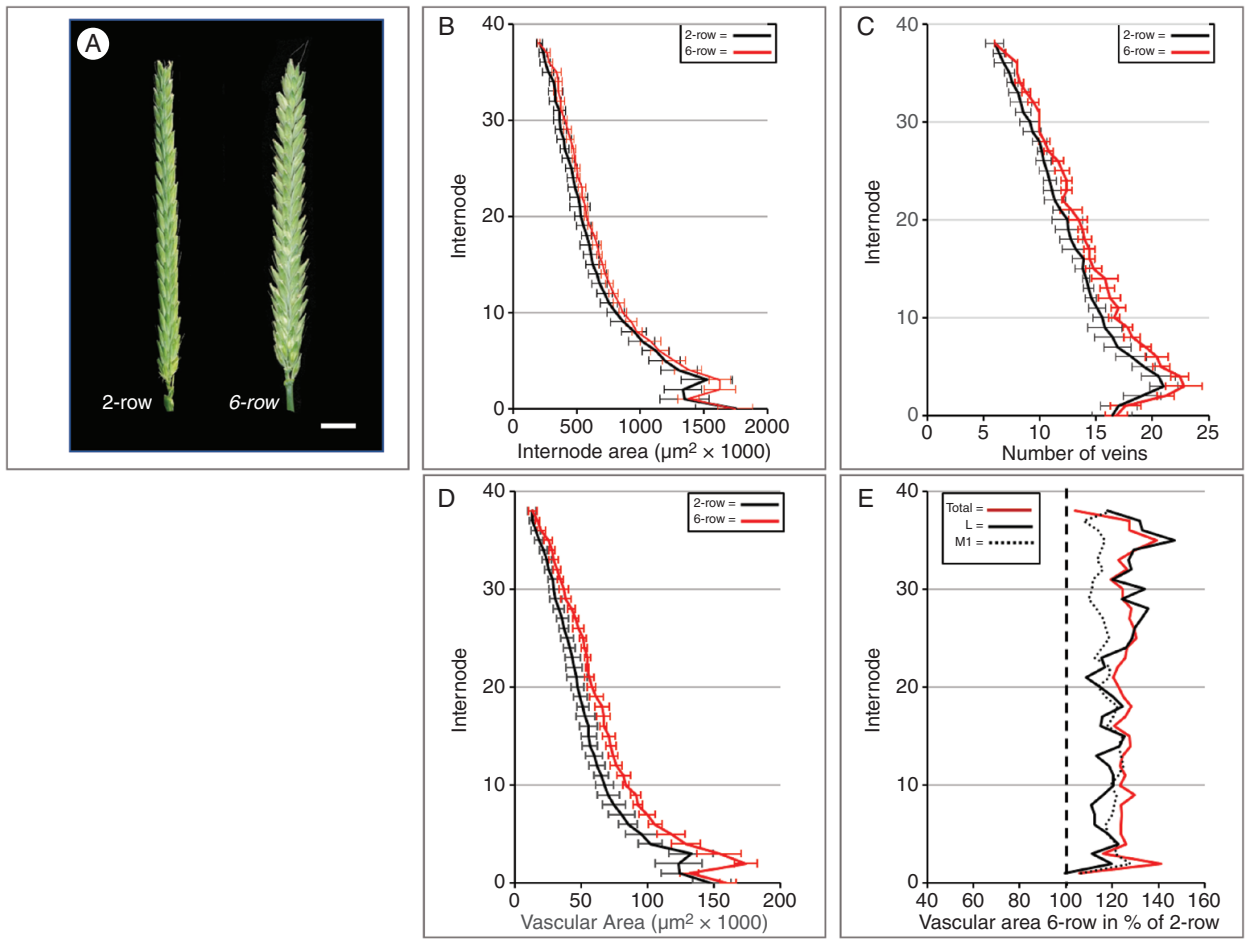
Floret fertility and rachis vasculature. (A) Spike phenotype of two-rowed GP and isogenic six-rowed GP*-vrs1*. (B) Two-rowed GP and six-rowed GP*-vrs1* have similar internode areas along the spike. Even when extra-numeral veins are excluded, the six-rowed rachis contains more veins (C) and has a distinctly larger total vascular area (D) than the two-rowed rachis. (E) Against the normalized values for the two-rowed GP to 100 % (straight vertical dotted line), the significant increase in total vascular area of the six-rowed GP*-vrs1* (solid red line) is caused by a general increase in vein size as shown for laterals (solid black line) and first-order median veins (dotted black line). For matters of clarity, standard deviation bars were omitted. Scale bar: 1 cm (A).

**Fig. 11.**
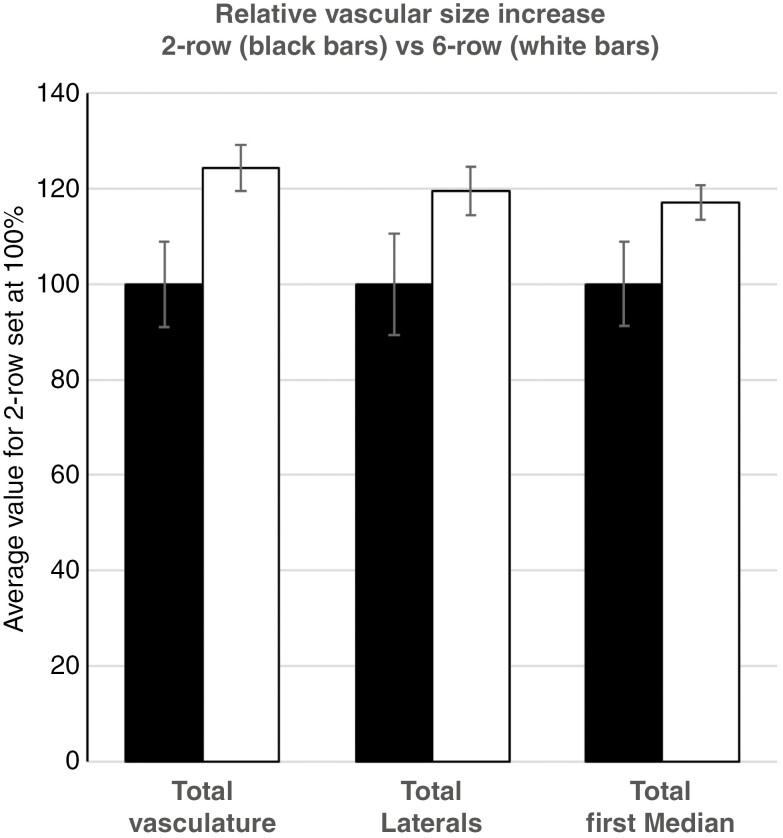
Increase in total vascular area and area of individual vein orders upon conversion of the two-rowed GP (solid black bars, *n* = 9) into the isogenic six-rowed GP-*vrs1* (open black bars, *n* = 5). Results indicate that the increase in total vein area is caused predominantly by an increase in vein size.

Transmutation of the two-rowed GP into the isogenic six-rowed GP*-vrs1* (Fig. 10A) triples the number of fertile spikelets per node without changing the number of spikelets per se (Komatsuda *et al.*, 2007; Zwirek *et al.*, 2018). Despite this conversion, two-rowed GP (spike size 36.9 ± 0.9 nodes, *n* = 9) and six-rowed GP*-vrs1* (spike size 37.2 ± 0.7 nodes, *n* = 5) spikes with a similar number of nodes display virtually identical internode areas along the rachis (Fig. 10B). Differences appear to reside solely in the vasculature. Including all veins in the comparison, the rachis of the six-rowed GP*-vrs1* (14.8 ± 0.2 veins per internode) displays a small but significant increase in the number of veins compared with the two-rowed GP (13.1 ± 0.9 veins per internode). Even when discounting the large number of putatively spikelet-derived extra-numeral veins in between lateral and first-order median veins, the difference in the remaining number of veins between two-rowed GP (12.8 ± 0.7 veins per internode) and six-rowed GP*-vrs1* (14.0 ± 0.1 veins per internode) remains significant (Fig. 10C). This ~10 % increase in the number of veins in the six-rowed GP*-vrs1* is accompanied by an even larger increase in vascular area. Excluding the extra-numeral veins, the average total vascular area per internode in the six-rowed GP*-vrs1* is substantially (26.2 %) larger than that in the two-rowed GP (Fig. 10D). Analysis of lateral and first-order median veins confirms that the transformation into a six-rowed phenotype goes along with a marked increase in the dimensions of individual veins, including an average 21.7 % increase in area for lateral veins and a 17.4 % increase in area for first-order median veins (Figs 10E and 11). This identifies expansion of vascular size as a main cause for the rise in the vascular area in the six-row GP*-vrs1*.

### Extra-numeral veins in two-rowed and six-rowed rachises

**Fig. 12.**
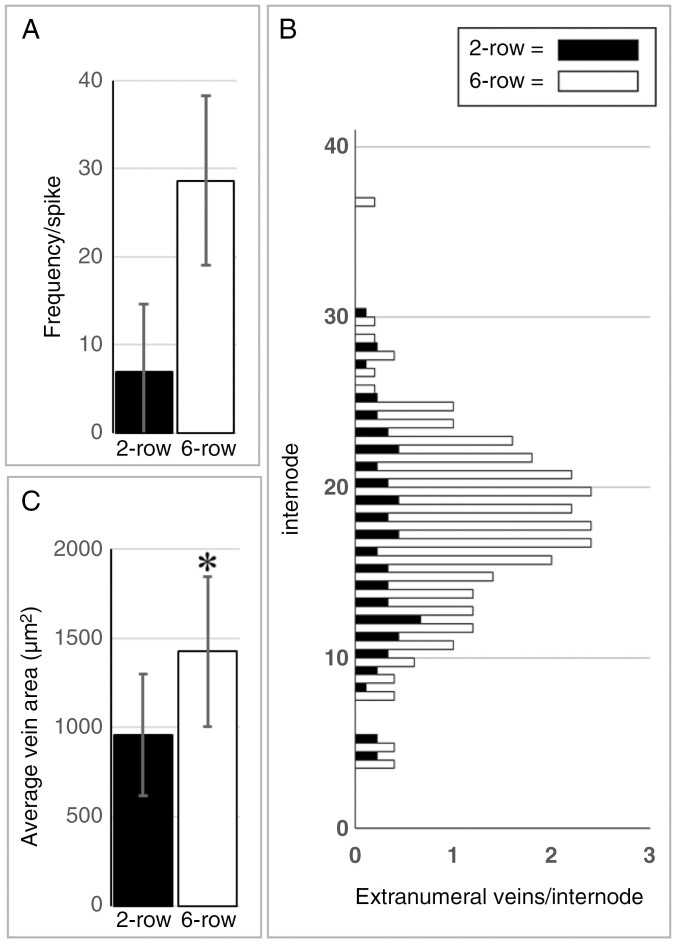
Peripheral extra-numeral veins in two-rowed GP (spike size 36.9 ± 0.9 nodes, *n* = 9) and isogenic six-rowed GP*-vrs1* (spike size 37.2 ± 0.7 nodes, *n* = 5). (A) The frequency of extra-numeral veins in the six-rowed GP*-vrs1* is about four times higher than that in the two-rowed GP. (B) Distribution analysis shows that in both two-rowed GP and six-rowed GP*-vrs1*, extra-numeral veins occur predominantly in the middle part of the spike. (C) The average area of the six-rowed extra-numeral veins (*n* = 142) is significantly larger (*P* > 0.001) than that of the two-rowed extra-numeral veins (*n* = 62).

The subgroup of extra-numeral veins located between lateral and median veins in the two-rowed GP rachis appears to originate from the lateral spikelets. A serial internode section analysis of the six-rowed GP*-vrs1* supports this assumption. Furthermore, given that there was never more than one extra-numeral vein between a lateral and first-order median vein, it seems that each spikelet is connected to the rachis by a single extra-numeral vein. As in the two-rowed GP, the number of extra-numeral veins in rachises of six-rowed GP*-vrs1* varies substantially between individual spikes (Fig. 12A). Nevertheless, the distribution pattern along the rachis is similar between two-rowed GP and six-rowed GP*-vrs1* (Fig. 12B). Compared with the two-rowed GP, the six-rowed GP*-vrs1* contains, on average, about four times as many extra-numeral veins (Fig. 12A, B), which are also substantially larger (Fig. 12C; *P* > 0.001). Furthermore, of the extra-numeral veins entering the peripheral part of the rachis, only ~50 % (21 of 41) may reach the next basipetal node in the rachis of two-rowed GP, compared with >70 % (60 of 83) in the rachis of six-rowed GP*-vrs1*.

### Spikelet reduction and rachis vasculature

**Fig. 13.**
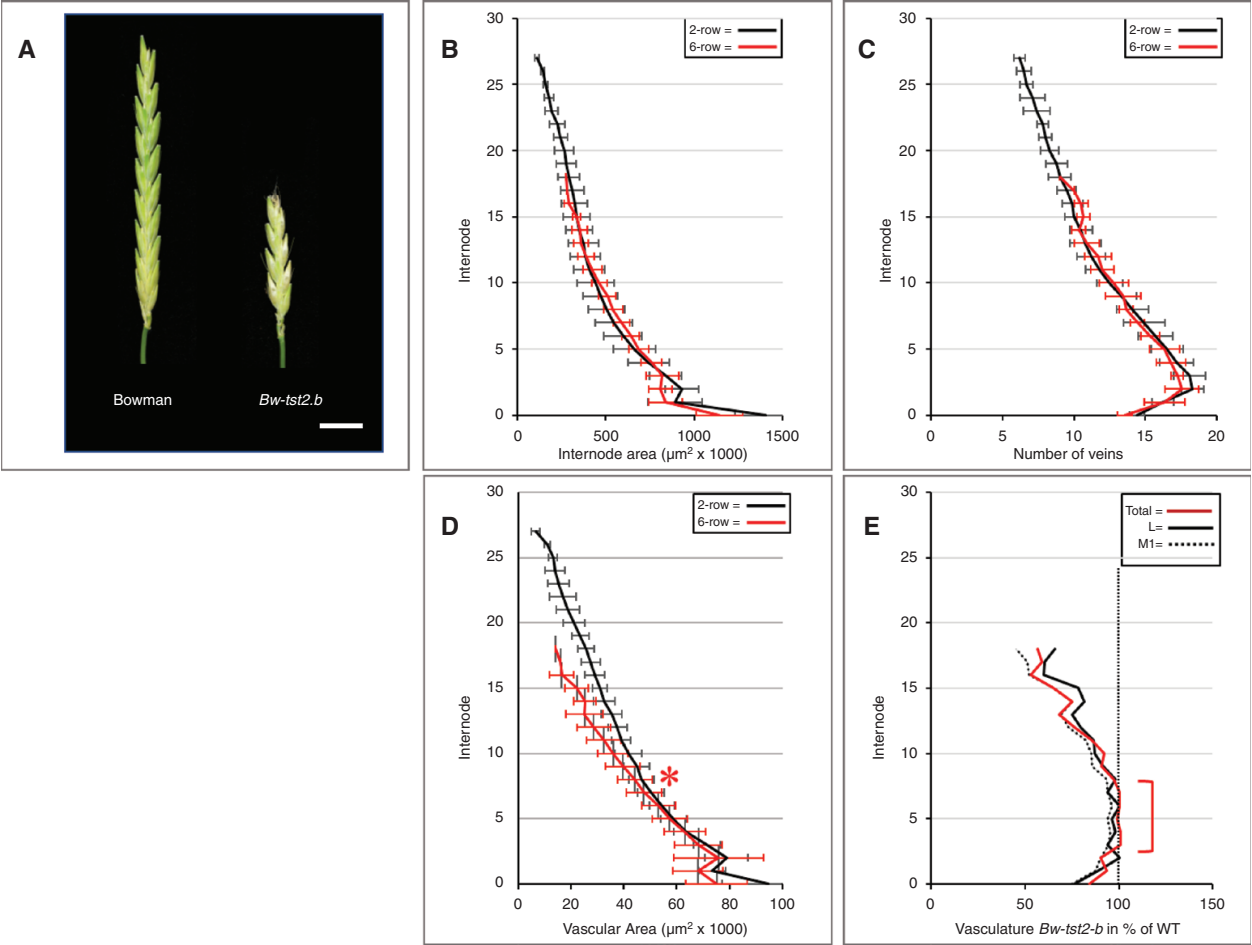
Effect of pre-anthesis tip degeneration on rachis vasculature. (A) Mature spikes of cv. Bowman and near-isogenic mutant BW-*tst2.b*. (B, C) Extensive pre-anthesis tip degeneration in the *tst2-b* mutant has no clear effect on internode area (B) or vascular number (C). (D) In the extreme base and from about internode no. 8 onwards, the total vascular area of the *tst2-b* mutant is distinctly less than that in cv. Bowman (* in D). (E) Vascular inequalities between cv. Bowman and BW-*tst2.b* involve all vein classes to the same extent. Against the normalized values for cv. Bowman to 100 % (straight vertical dotted line), in BW-*tst2.b* the total vascular area (red line) and the area of laterals (black line) and first-order median veins (dotted black line) are significantly reduced at the extreme base, nearly similar between internode 3 and 8 (brace) and become progressively smaller again beyond internode 8. For matters of clarity, standard deviation bars were omitted in E. Scale bar: 1 cm (A).

Barley plants exhibiting the *tst2.b* mutation undergo extended pre-anthesis tip degeneration, resulting in a significantly shortened spike (Dahleen *et al.*, 2007; Huang *et al.*, 2023). To examine the effects on the rachis vasculature, spikes of cv. Bowman (spike size 25.3 ± 1.8 nodes, *n* = 11) were compared with those of the near-isogenic mutant BW*-tst2.b* (spike size 14.2 ± 1.8 nodes, *n* = 13) (Fig. 13A). Despite the severe reduction in spike size, the *tst2.b* mutation has no effect on either internode area or the number of veins in the rachis (Fig. 13C). Concerning total vascular area, two main regions of disparities between wild-type and *tst2.b* exist (Fig. 13D): the first is a short region at the extreme base of the rachis, while the second region starts and becomes progressively pronounced from about internode 8 onwards. These deviations become more evident when data for the total vascular area, lateral veins and first-order median veins are compared. The results show that all vascular components of the *tst2.b* mutant are affected to the same extent in the extreme base and progressively so from internode 8 onwards (Fig. 13E). In the rachis section running from internode 3 to 8, however, the total vascular area and size of individual veins do not differ between the wild-type and mutant (Fig. 13E).

### Rachis parameters in wild barley

**Fig. 14.**
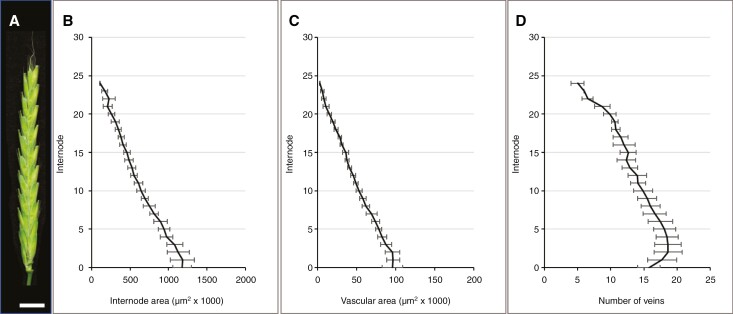
Dynamics of internode parameters along the rachis of wild barley (*Hordeum spontaneum*) acc. HID003. Data were collected from mature spikes between 20 and 24 nodes in size (average 22.0 ± 1.4 nodes, *n* = 8). (A) Mature spike. (B, C) Both internode area (B) and vascular area (C) display near-linear declines along the rachis. (D) Vascular number reaches a maximum value around internode 3 before gradually declining towards the tip. Scale bar: 1 cm (A).

A comparative analysis of wild barley accession HID003 (average spike size 22.0 ± 1.4 nodes, *n* = 8) shows that its vascular layout is similar to that of the barley two-rowed GP and cv. Bowman, with the number of main vascular bundles reaching a peak around internode 3 (compare Figs 2, 13 and 14).

**Fig. 15.**
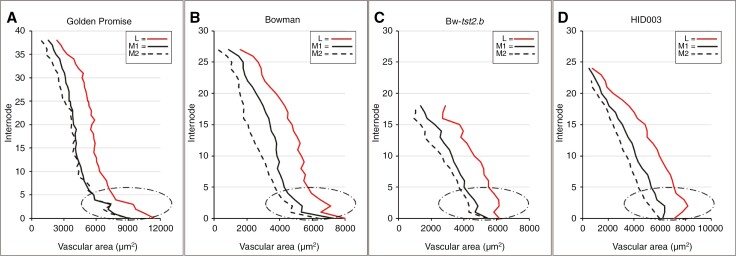
Differences in the dynamics of individual vein orders between cv. Golden Promise (A), cv. Bowman (B), BW-tst2.b (C) and wild barley accession HID003 (D). At the base of the rachis, vascular bundles in cv. Golden Promise and cv. Bowman decline steeply in size, whereas in mutant BW*-tst2.b* and wild barley accession HID003 the decline is more linear, sometimes even displaying a maximum at internode 2 or 3. For matters of clarity, standard variations were omitted. Abbreviations: L, lateral vein; M1, first-order median vein; M2, second-order median vein.

There are slight differences in the dynamics of the internode area and vascular area. In two-rowed GP and Bowman, these parameters show more or less distinct two-stepped declines (Figs 2A, B and 13B, D), whereas the declines in HID003 are near linear (Fig. 14B, C), thus resembling the profiles seen in BW-*tst2.b* (Fig. 13B, D). Also, regarding the dynamics of individual vein orders, HID003 appears more similar to BW-*tst2.b* (Fig. 15). Instead of a clear two-stepped decline in size as seen in the two-rowed GP and Bowman (Fig. 15A, B), veins in HID003 and BW-*tst2.b* display more linear declines (Fig. 15C, D).

## DISCUSSION

Barley is considered to be a sink-limited cereal, in which assimilate supply exceeds the storage capacity of the grains (Bingham *et al.*, 2007). Also, transport capacity of the rachis vasculature is in excess of the demands during grain filling (Yang and Zhang, 2006). This apparent absence of bottle-neck features might explain why the barley rachis vasculature has received little research attention in the past near half-century. As a follow-up to the pivotal study by Kirby and Rymer (1975) on barley rachis vasculature, the main aim of the present work was to clarify the relationship between vasculature and spike size, to define vascular dynamics and to provide an improved model for overall vascular organization within the rachis.

### Vascular parameters and spike size

The transition from a shoot to a spike phenotype takes several internodes to materialize (Supplementary Data Video). This also accounts for the vascular organization. Although often implied (Percival, 1921; Whingwiri *et al.*, 1981), features such as vascular number and size do not go through a gradual linear decline along the rachis (Fig. 2). Most conspicuous is that the number of main vascular strands displays an absolute maximum around internode 3–4 irrespective of the source material used (Figs 2C, 10C, 13C and 14D). This might unveil the vein initiation site within the early rachis, from which veins develop both acropetally into the apex and basipetally into the culm. Although Kirby and Rymer (1975) situated this site between spikelet primordium 6 and 10, observations by Pizzolato (1997) suggest a much more basal location.

The morphogenesis of the barley rachis vasculature appears to be the result of two distinct processes: initiation and processing. The basic layout of the spike, including that of the rachis vasculature, is concluded by the time of maximum yield potential, and differentiation of the initiated organs is finished by anthesis (Waddington *et al.*, 1983; Thirulogachandar and Schnurbusch, 2021). Using mature spikes at the time of grain filling, we accordingly found the number of veins to be correlated only weakly with spike size (Fig. 4C), whereas internode area and total vascular area were strongly correlated (Fig. 4A, B). Correlations between vascular parameters and internode area, however, appeared very stable and unrelated to spike size (Fig. 5A–C), for which reason the factor internode area can be used for a comparative analysis of transport capacity between individual spikes (Rosell *et al.*, 2017).

### Vascular organization and transport efficiency

Transport resistance is directly proportional to the length of a vessel, but inversely proportional to the radius to the fourth power (Tyree and Ewers, 1991; Cruiziat *et al.*, 2002; Chavarria and Pessoa dos Santos, 2012). This makes vessel size a central factor in transport efficiency, with small changes having strong effects (Rosell *et al.*, 2017). The strong correlation between the internode area and vascular parameters (Figs 4 and 5) confirms that the transport capacity of the rachis is modelled mainly by vessel size. This emphasis on transport efficiency is also reflected in the organization and dynamics of lateral and median vein orders (Fig. 6). During spike development, new veins are initiated inwards of already existing ones (Kirby and Rhymer, 1975). Measurements of individual vein orders (Fig. 6C) not only suggest a near-uniform size for all fully developed median veins (Fig. 6) but also indicate that the initiation of a new order of veins coincides with the previous order reaching maturity (Fig. 6C).

It is tempting to speculate that the termination of the inflorescence meristem around the awn primordium stage not only determines the maximum yield potential (Thirulogachandar and Schnurbusch, 2021), but also puts a hold on any further vascular development, freezing the vascular bundles of the rachis in their respective stages of initiation. As such, the central area of the barley rachis appears to be occupied by a staggered array of small, putative immature veins (Fig. 6D). Given that marks of immaturity include the absence or incompleteness of a bundle sheath (Esau, 1943), the central rachis might thus be a stockpile of potentially leaky veins suited to providing a continuous supply of nutrients and metabolites along the central axis of the rachis. In contrast, the large mature veins in the rachis periphery might serve a transport role, because their constant dimensions should mitigate long-distance transport resistance and facilitate apical supply. Observations on the uptake and distribution of labelled sucrose in the barley spike by Melkus *et al.* (2011) showed signals accumulating in the central area of the rachis, i.e. at the spikelet attachment site. This supports our hypothesis of a spatial separation between bulk transport taking place in the rachis periphery while local accumulation and delivery to the spikelets occurs in the centre of the rachis.

### Interaction between spikelet and rachis vasculature

Once veins have reached the centre of the rachis, they start to diminish in size (Fig. 6) and ultimately end near or at a node (Fig. 7). The centre of the rachis is also one of two locations where presumed spikelet-derived extra-numeral veins enter, with the other location being in the periphery, between lateral and first-order median veins (Fig. 8). Our observations on two-rowed and six-rowed barley suggest that from each spikelet only a single vein grows into the rachis. Once in the rachis, these veins, by analogy with leaf veins, probably extend basipetally and ultimately fuse with the rachis vasculature (Hitch and Sharman, 1968; Nelson and Dengler, 1997; Pizzolato, 1997). Through fusion events, such as shown in Fig. 9C, the declining veins occupying the centre of the rachis might, over the course of several nodes, be connected to multiple spikelet-derived veins. The latter might eventually ‘cap off’ the rachis vein, thus giving the impression of these veins ending at a node (Fig. 7).

Not all extra-numeral veins will merge with the rachis vasculature, which is the reason spikelet-derived veins become visible in the first place (Figs 7C and 9A, B). This failure to fuse is especially evident in the rachis periphery (Fig. 8B) and is particularly prominent in the six-rowed GP-*vrs1* line (Fig. 11). Given that most fusion events observed took place in the centre of the rachis, it is tempting to speculate that this might reflect an overall inability of spikelet veins to merge with the large mature veins of the rachis periphery, whereas they might fuse readily with the small immature veins occupying the rachis centre. The high incidence with which, in the six-rowed GP-*vrs1*, veins originating from the lateral spikelets do not fuse with the rachis vasculature also calls into question the relevance of vascular continuity between the spikelet and rachis vasculature. Kirby and Rymer (1974) remarked that when rachis veins transverse a node, i.e. the main place for nutrient exchange, it can be near to impossible to distinguish these veins from the surrounding parenchymal tissue rich in putative transfer cells. The facultative character of vascular continuity suggests that both vascular systems might fulfil their roles independently, with the rachis veins delivering nutrients to the node and the spikelet veins collecting nutrients from the node. A fusion between the two vascular systems near or at the node might then be no more than a non-essential coincidence. Although vascular discontinuity also exists in the floral axis (Zee and O’Brien, 1970; Kirby and Rymer, 1975), it will require a three-dimensional reconstruction to elucidate the true interaction between rachis and spikelet vasculature.

### Floret fertility and rachis vasculature

The events that distinguish a two-rowed GP and a six-rowed GP*-vrs1* establish themselves after the stage of maximum yield potential (Zwirek *et al.*, 2018; Thirulogachandar and Schnurbusch, 2021), i.e. after the basic layout of the rachis, including its vasculature, has been concluded. In accordance with this, the transmutation of the two-rowed GP into a six-rowed GP*-vrs1* did not affect the surface area of the rachis per se. Excluding the increase in extra-numeral veins (Fig. 12), the number of main rachis veins rose by ~10 % (Fig. 10C). Far more consequential, however, is the increase in vein size by ~20 % (Figs 10E and 11). Concerning the fixed ratio between the dimensions of vascular bundles and that of the metaxylem vessels (Fig. 5), a 20 % increase in vessel area (*r*^2^ × π) should, according to the Hagen–Posseuille equation [*V*/*t* = (*r*^4^ × π × Δ*P*)/(8 × η × *linwhich* in which V = volume, t = time, r = radius of vessel, Δ*P* = pressure difference between two ends of a vessel, η = viscosity, l = length of vessel], suffice to increase the xylem transport capacity of the six-rowed GP*-vrs1* rachis by >40 %. It thus seems that spikelet/floret fertility in the *vrs1* mutant has a quantitative effect on transport capacity along the whole rachis. Given that Sakuma *et al.* (2013) discovered *Vrs1* to be localized in the vasculature of the developing rachis, this protein is likely to play a role in vascular development.

To examine possible consequences of the opposite, i.e. a reduction in spikelet/floret fertility, the cv. Bowman was compared with its near-isogenic line, BW*-tst2.b*, showing extended pre-anthesis tip degeneration (Fig. 13A) attributable to a mutation in a vascular-expressed *CCT MOTIF FAMILY4* (*HvCMF4*) gene (Huang *et al.*, 2023). This mutation reveals itself after maximum yield potential (Huang *et al.*, 2023) and mainly affects the dimensions of the veins (Fig. 13D), while the internode area and number of veins remain unaltered (Fig. 13B, C). The vascular modifications involve all veins in a similar fashion (Fig. 13E). Far from being uniform along the rachis, the effect was pronounced in the extreme base, near to absent between internodes 3 and 8 and from then on becoming progressively stronger again towards the apex (Fig. 13D, E). In the last internode, average vein area had decreased by ~40 % (Fig. 13E), reducing the xylem transport capacity by >60 % in comparison to the wild-type. This not only supports observations by Huang *et al.* (2023) implying that an altered vasculature pre-dates pre-anthesis tip degeneration, but also underlines the importance of histological data for our understanding of the physiological functions of the barley spike.

### Rachis vasculature in wild barley

In all barley plants studied here, vascular number peaked slightly above the rachis base, identifying this as the likely vein initiation site. Although the decline in total vascular area and that of individual vein orders along the rachis is clearly two-stepped in cv. Golden Promise and cv. Bowman, it is more linear in wild barley acc. HID003 and mutant BW-*tst2.b* (Figs 2B, 13D, 14C and 15). Given that altered vascular dynamics are likely to have consequences for the transport efficiency in the barley rachis, this feature deserves further attention to establish whether it might be correlated with traits such as spike size, grain yield or even domestication itself. As work by Huang *et al.* (2023) has shown, single alleles can be responsible for increasing the vascular dimensions in both the base and apical region of a spike, thus greatly affecting spike yield.

## Conclusion

The morphogenesis and organization of the rachis vasculature is a multi-step process. In the first phase, the basic layout is established, featuring the ultimate number of main rachis veins (Waddington *et al.*, 1983; Thirulogachandar and Schnurbusch, 2021). This is followed by a second phase, in which the dimensions of the rachis veins and, hence, the transport capacity of the rachis as a whole are adjusted to accommodate future demands. In this, one of the determining factors appears to be spikelet fertility. Within the rachis vasculature, we can differentiate between mature veins in the lateral periphery, in charge of long-distance transport, and a staggered array of immature veins in the centre, responsible for local spikelet supply (Figs 9 and 15). The centre of the rachis also serves as the main port of entry for spikelet-derived veins; the other positions are located in the rachis periphery (Figs 10 and 11). The frequently observed failure of spikelet-derived veins to merge with the rachis vasculature indicates that vascular continuity between the spikelets and rachis might be a non-essential feature.

To summarize, the analysis of transverse internode sections, used here to unravel the basic functional characteristics of the barley rachis vasculature, is a simple method that should also be useful to investigate possible correlations between vascular capacity and other major plant traits.

## SUPPLEMENTARY DATA

Supplementary data are available at *Annals of Botany* online and consist of the following.

Video: serial transverse internode sectioning through the rachis of a barley spike cv. Golden Promise.

mcae023_suppl_Supplementary_infornation_Video
